# Boron rich nanotube drug carrier system is suited for boron neutron capture therapy

**DOI:** 10.1038/s41598-021-95044-0

**Published:** 2021-07-30

**Authors:** Fabian Heide, Matthew McDougall, Candice Harder-Viddal, Roy Roshko, David Davidson, Jiandong Wu, Camila Aprosoff, Aniel Moya-Torres, Francis Lin, Jörg Stetefeld

**Affiliations:** 1 Department of Chemistry, University of Manitoba, Winnipeg, MB R3T 2N2 Canada; 2 Department of Chemistry and Physics, Canadian Mennonite University, Winnipeg, MB R3P 2N2 Canada; 3 Department of Physics and Astronomy, University of Manitoba, Winnipeg, MB R3T 2N2 Canada; 4 Institute of Biomedical and Health Engineering, Shenzhen Institutes of Advanced Technology, Chinese Academy of Sciences, Shenzhen, 518055 China

**Keywords:** Biochemistry, Carrier proteins, Targeted therapies

## Abstract

Boron neutron capture therapy (BNCT) is a two-step therapeutic process that utilizes Boron-10 in combination with low energy neutrons to effectively eliminate targeted cells. This therapy is primarily used for difficult to treat head and neck carcinomas; recent advances have expanded this method to cover a broader range of carcinomas. However, it still remains an unconventional therapy where one of the barriers for widespread adoption is the adequate delivery of Boron-10 to target cells. In an effort to address this issue, we examined a unique nanoparticle drug delivery system based on a highly stable and modular proteinaceous nanotube. Initially, we confirmed and structurally analyzed *ortho*-carborane binding into the cavities of the nanotube. The high ratio of Boron to proteinaceous mass and excellent thermal stability suggest the nanotube system as a suitable candidate for drug delivery into cancer cells. The full physicochemical characterization of the nanotube then allowed for further mechanistic molecular dynamic studies of the *ortho*-carborane uptake and calculations of corresponding energy profiles. Visualization of the binding event highlighted the protein dynamics and the importance of the interhelical channel formation to allow movement of the boron cluster into the nanotube. Additionally, cell assays showed that the nanotube can penetrate outer membranes of cancer cells followed by localization around the cells’ nuclei. This work uses an integrative approach combining experimental data from structural, molecular dynamics simulations and biological experiments to thoroughly present an alternative drug delivery device for BNCT which offers additional benefits over current delivery methods.

## Introduction

Boron neutron capture therapy (BNCT) is a potentially promising treatment for malignant tumors^1^, especially for head and neck cancer, colorectal cancer and melanoma^2,3^. The technology is based on the nuclear capture reaction of thermal (low energy < 0.5 eV, limited depth of penetration) and epithermal neutrons (> 0.5 eV < 10 keV, greater depth of penetration) using nonradioactive ^10^B, which causes the production of a ^7^Li particle and an α particle with ~ 2.3 MeV thermal energy^1,4^. The high linear energy transfer of charged particles is accompanied by a short spatial trajectory of 5–10 µm, the approximate diameter of one cell. As a consequence, radiation damage is strictly confined to cells containing ^10^B particles minimizing damage on surrounding tissue. BNCT has thus been described as a two-step process which combines two low toxicity procedures, ^10^B delivery and activation by neutron radiation, to precisely eliminate the targeted cells.

For widespread adoption, several important questions remain to be addressed in the development of BNCT as a clinical treatment. First, a carrier is required that allows for the selectively delivery of the isotope ^10^B isotope to tumor cells. The fact that BNCT is a spatially limited non-invasive therapeutic approach is a major advantage of the technique. However, this implies that the boron compounds have to be placed either in close spatial vicinity to or, more preferably, inside the tumor cell to maximize dosage to the cell’s nucleus. Second, to ensure effective neutron radiation treatment, delivery amounts of > 20 µg ^10^B/ g tumor are required^5^. Additionally, delivery methods need to meet specific requirements such as high tumour/tissue uptake ratios, low toxicity and rapid clearance from the patient’s body^1^. Considerable research has been focused on developing carrier systems which are capable of targeting cancer cells for BNCT. These include lipid based nanocarriers^6^, inorganic nanoparticles^7–9^, drug conjugates^10^ and more recently, peptide based carrier systems^11,12^. In general, nanoparticles can offer certain advantages over non-conjugated compounds, such as overcoming drug solubility issues, increased chemical stability, decreased toxicity and improved distribution with potential tissue-specific targeting^7^. However, in order to present a novel nanoparticle for the use of drug delivery, the physicochemical properties have to be closely examined. Ideally, binding mechanisms and targeting are demonstrated which endorse further studies.

The development of effective targeting strategies and the sufficient upload of ^10^B isotopes are urgent problems in the area of BNCT research. Here, we present an unconventional drug delivery device entitled Right-Handed Coiled Coil-Nanotube (RHCC-NT) which is ideally suited to uptake boron clusters into its large internal cavities. In addition to binding small molecules, this nanotube can be internalized by eukaryotic cells, which offers possibilities for targeted drug delivery in BNCT. RHCC-NT was first described as an S-layer protein component of the archaea *Staphylothermus marinus*^13^. It has been shown to possess highly unusual properties in respect to cargo uptake^14–17^, targeted drug delivery^18–20^ and robustness^13,21^. Our study focuses on an integrated approach combining structural biology, biophysical and in vitro functional assays to characterize RHCC-NT on physicochemical and biological levels as a unique drug delivery system for targeted ^10^B delivery. In addition to this, mechanisms and free energy profiles were simulated by molecular dynamics simulations to understand the protein nanotube’s solution behaviour and ligand uptake into the cavities on a molecular level. Our data demonstrate that RHCC-NT can uptake *o*-carboranes, C_2_B_10_H_12_, and deliver them into tumor cells for the use in BNCT.

## Materials and methods

### Expression of RHCC-NT

The expression and purification methods of Right-Handed Coiled Coil Nanotube (RHCC-NT) have previously been described^14^. In summary, the RHCC-NT gene was cloned in a pET15b vector and transformed into *Escherichia coli* BL21 (DE3). Positive clones were selected with ampicillin and used to inoculate liquid LB ampicillin for protein production. His-tagged RHCC-NT expression was induced with Isopropyl β-D-1-thiogalactopyranoside. After expression of RHCC-NT, the cells were pelleted and re-suspended in binding buffer (20 mM TRIS pH 8.0, 5 mM Imidazole, 500 mM NaCl, 8 M Urea) and kept on ice until cell lysis by sonication. Purification of the His-tagged RHCC-NT was achieved by affinity chromatography on Ni^2+^-Sepharose (GE Healthcare) as previously described^13^. The His-tag was removed by thrombin cleavage at 50 °C and RHCC-NT was purified by anion-exchange chromatography on a HiTrap Q Sepharose (GE Healthcare) column. Lastly, RHCC-NT was dialyzed into 20 mM TRIS pH 8.0, 154 mM NaCl and stored at 5 °C until further use.

### Boron nuclear magnetic resonance (NMR) spectroscopy

RHCC-NT was concentrated to 11.0 mg/mL in 20 mM TRIS pH 8.0, 154 mM NaCl using a Centrifugal Filter Concentrator, MWCO 3 kDa (Amicon) after which 2 mL of solution were incubated with 10 mg of *o*-carborane, C_2_B_10_H_12_ for 1 week at 50 °C. Additionally, control samples consisting of 2.5 mg *o*-carborane in 20 mM TRIS pH 8.0 154 mM NaCl and 2.5 mg *o*-carborane in toluene (100%, High Performance Liquid Chromatography grade) were prepared 18 h before NMR measurements. All samples were filtered through a 0.1 μm centrifuge filter tube serving the purpose to separate free *o*-carborane grains from the sample solution. 550 µL samples supplemented with 10% (v/v) deuterium oxide and 5 µM 4,4-dimethyl-4-silapentane-1-sulfonic acid were prepared, dispensed into a quartz NMR tube and measured in a Bruker Avance III 500 MHz NMR spectrometer at 25 °C utilizing a 5 mm room-temperature BBFO probe. ^11^B NMR spectra were collected with power-gated ^1^H decoupling acquiring 65,000 points, totaling 1024 transients per spectrum with a 2 s recycle delay. Spectra were processed in TopSpin 3.5 pl 6 (Bruker NMR Software) with removal of the first 44 points in each free induction decay (FID) and replacement via linear back-prediction based on 256 components in order to reduce ^11^B background signals from probe materials.

### Dynamic light scattering

The size distributions of native RHCC-NT and RHCC-NT incubated with *o*-carborane were compared using dynamic light scattering for quality control (Figure S1). Protein contents were concentrated to 10 mg/mL using a Centrifugal Filter Concentrator, MWCO 3 kDa (Amicon). Samples were filtered through a 0.1 μm centrifuge filter tube after which particle size distributions of RHCC-NT samples were measured in triplicates at 20 °C using a Zetasizer Nano ZS90.

Further, the size distribution and thermal stability of RHCC-NT was measured over a thermal range from 20 to 95 °C using a NanoTemper Prometheus Panta (Figure S2/S3). Protein samples were measured in triplicates at 10 mg/mL protein concentration.

### RHCC-NT carborane complex crystallization

RHCC-NT was incubated with *o*-carborane, C_2_B_10_H_12_ at 50 °C for 1 week after which the structural integrity was tested using Dynamic Light Scattering. RHCC-NT was dialyzed into 10 mM TRIS pH 7.5, 154 mM NaCl and concentrated to 10 mg/mL. Crystals were set up in a vapour diffusion crystallization experiment in 100 mM ammonium acetate (C_2_H_7_NO_2_), 50 mM sodium cacodylate trihydrate ((CH_3_)_2_AsO_2_Na·3H_2_O) pH 6.5, 10% 2-propanol (C_3_H_8_O), 15 mM magnesium acetate tetrahydrate ((CH_3_COO)_2_ Mg·4H_2_O). Rod shaped crystals formed after 1 week. Data was collected on a Rigaku rotating anode MM-007HF diffractometer with a wavelength of 1.54178 Å in 1° wedges at 100 K. Diffraction data was processed with XDS and the CCP4-package. Phases were calculated in Phaser using a RHCC-NT search model (PDB: 1YBK) and the *o*-carborane stereochemical restraints were generated using eLBOW (Phenix package). The structures were built manually and refined crystallographically using the Coot suite and the Phenix software package.

The crystallography data table can be found in the SI document (Table S1).

### Molecular dynamics simulations

Molecular dynamics simulations were performed with the GROMACS molecular dynamics simulation package^22^ using the AMBER force field parm94, and the TIP3P water model. All simulations were performed on a RHCC-NT-*o*-carborane (C_2_B_10_H_12_) complex in which all four cavities were simultaneously occupied by a single carborane molecule. The complex was formed by inserting a modeled structure of an *o*-carborane molecule into each cavity of the measured structure of an RHCC-NT tetramer (PDB code 1FE6)^13^ which was crystallized at T = 298 K. The Amber 99 force field parameters for *o*-carborane were obtained from literature^23–25^. Each RHCC-NT complex was solvated in water and Na^+^ ions to neutralize the system. The measured structure was energy minimized using the method of steepest descent. Potential pathways for the shuttling of *o*-carborane between one of the cavities of RHCC-NT and the solvent were identified using the combined methods of steered molecular dynamics^26–30^ and umbrella sampling^31–33^.

Additional details can be found in the SI document (Table S2).

### RHCC-NT cell assays

RHCC-NT was labeled with either Invitrogen Alexa Fluor 488 (AF488) or Invitrogen Alexa Fluor 647 (AF647) dye using amine reactive NHS ester crosslinking chemistry following the Invitrogen NHS Ester protocol. In short, RHCC-NT was dialyzed into PBS, pH 7.4 after which the amine reactive compounds were solubilized in DMSO to a final concentration of 5 mg/mL. The reactive dye solution was added to the protein sample using a 50× molar excess and allowed to react at room temperature for 1 h until reaction termination by addition of TRIS buffer, pH 7.5 to a final concentration of 100 mM. Fluorescently labeled protein samples were dialyzed in 20 mM TRIS pH 8.0, 154 mM NaCl, concentrated to 5 mg/mL and stored at 5 °C until experimental use.

MCF-7 cells were cultured from a frozen cryovial in a T75 flask (Thermo Fisher Scientific) at a seeding cell density of 1 × 10^5^ cells/mL. The cells were grown in Dulbecco's Modified Eagle Medium (DMEM), 5% Fetal Bovine Serum (FBS) at 37 °C, 5% CO_2_ in a humidified atmosphere until 90% confluency. Cells were split into new flasks and grown until 80% confluency after which RHCC-NT-AF488 was added to one flask at a final concentration of 5 µM, while the other flask served as a negative control. After 16 h of incubation, MCF-7 cells were washed twice with PBS and subjected to TrypLE Express Enzyme (Thermo Fisher Scientific) for cell detachment. Detached cells were centrifuged and re-suspended in DMEM. Cell samples for mammalian cell uptake of RHCC-NT were then analyzed by flow cytometry using a BD FACS Calibur flow cytometer (BD Biosciences, San Jose, CA, USA) which used a 488 nm laser and emission filter at 515/30 nm. Statistical analyses were performed on the fluorescent and scattering measurements for each cell population sample. Results are expressed as the median value and Coefficient of Variation (CV) over 10,000 events.

Further, MCF-7 cells were grown in well plates at a seeding density of 5 × 10^5^ cells/ml to 80% confluency. Varying concentrations of RHCC-NT-AF647 were added ranging from 1 nM to 10 µM. This also included a negative control. After an incubation period of 16 h at 37 °C, cells were washed, DAPI stained and imaged using an Invitrogen EVOS FL Auto 2 Imaging System (Thermo Fisher Scientific).

## Results

### Nanotube takes up *o*-carborane

**Figure 1:**
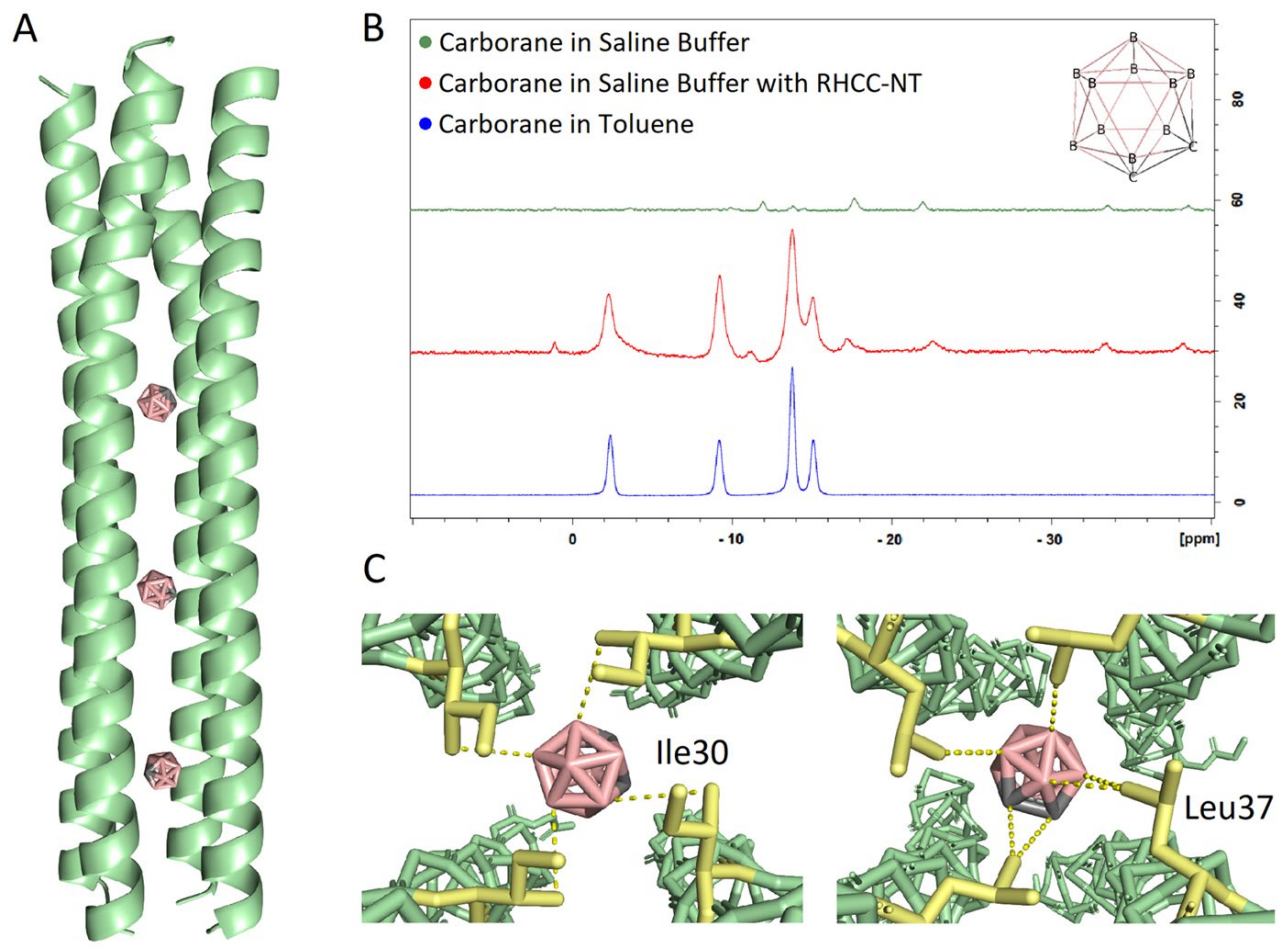
Nanotube-ligand structural models and uptake confirmation of *o*-carborane. (**A**) Structure of the RHCC-NT-C_2_B_10_H_12_ complex model at 2.2 Å (PDB ID 7R6H) which shows the uptake of three *o*-carborane molecules into the nanotube. (**B**) The uptake of *o*-carborane, C_2_B_10_H_12_ was confirmed using Boron NMR showing Boron shifts from an aqueous into a hydrophobic environment. (**C**) Models of *o*-carborane in the center cavity’s hydrophobic environment. The hydrophobic boron cluster is coordinated between the amino acid side chains of Ile30 and Leu37 from each helix via van der Waals forces. Protein figures were made using PyMOL 2.3.2 (https://pymol.org/2/).

Carborane, C_2_B_10_H_12_, a boron rich compound with applications in Boron Neutron Capture Therapy, binds into the hydrophobic cavities of RHCC-NT upon mutual incubation at 50 °C (Fig. 1A). To acquire structural information, we pre-incubated and crystallized the RHCC-NT-C_2_B_10_H_12_ complex which showed that the highly stable tetrameric proteinaceous nanotube, consisting of four right-handed helices, completely encloses the boron clusters (PDB ID 7R6H). We observed that the chemical interactions that stabilize the *o*-carborane inside the cavity are mainly hydrophobic, mediated by the amino acid side chains of Leu and Ile of the respective cavity. Based on spatial analysis, the two center cavities each have a spherical volume of around 240 Å^3^, allowing for binding of the *o*-carborane clusters with their steric bulk of 148 Å^3^^34^. The outer cavities have a spatial volume of around 180 Å^3^, surrounded by hydrophobic amino acid side chains which would also allow for *o*-carborane ligand binding. Nonetheless, based on experimental electron densities, we detected that three out of the four nanotube cavities contain boron clusters (Fig. 1A); *o*-carborane is absent in the N-terminal cavity of the nanotube. Thus, ligand incubation allows for an integration of 30 boron atoms per nanotube. Interestingly, the uptake of *o*-carborane did not cause any final conformational changes to the nanotube which is most likely due to the extreme rigidity of the macromolecule caused by its many intramolecular interactions such as salt bridges and additional hydrophobic interactions between the helices. Similar results have previously been shown for other hydrophobic ligands such as polycyclic aromatic hydrocarbons and sulfur rings^14,16^. To further confirm the uptake of *o*-carborane into RHCC-NT, we performed a Boron Nuclear Magnetic Resonance experiment comparing liganded versus unliganded nanotube samples. The Boron spectra under different conditions were measured, where the samples consisted of *o*-carborane in water, in toluene or in the presence of RHCC-NT. Our results show chemical shifts corresponding to the boron nuclei moving from a hydrophilic to a hydrophobic environment in the presence of the nanotube (Fig. 1B). These shifts validate the structural experiments and confirm diffusion of *o*-carborane into the inside of the nanotube upon incubation.

### Carborane uptake mechanism and associated free energies

**Figure 2:**
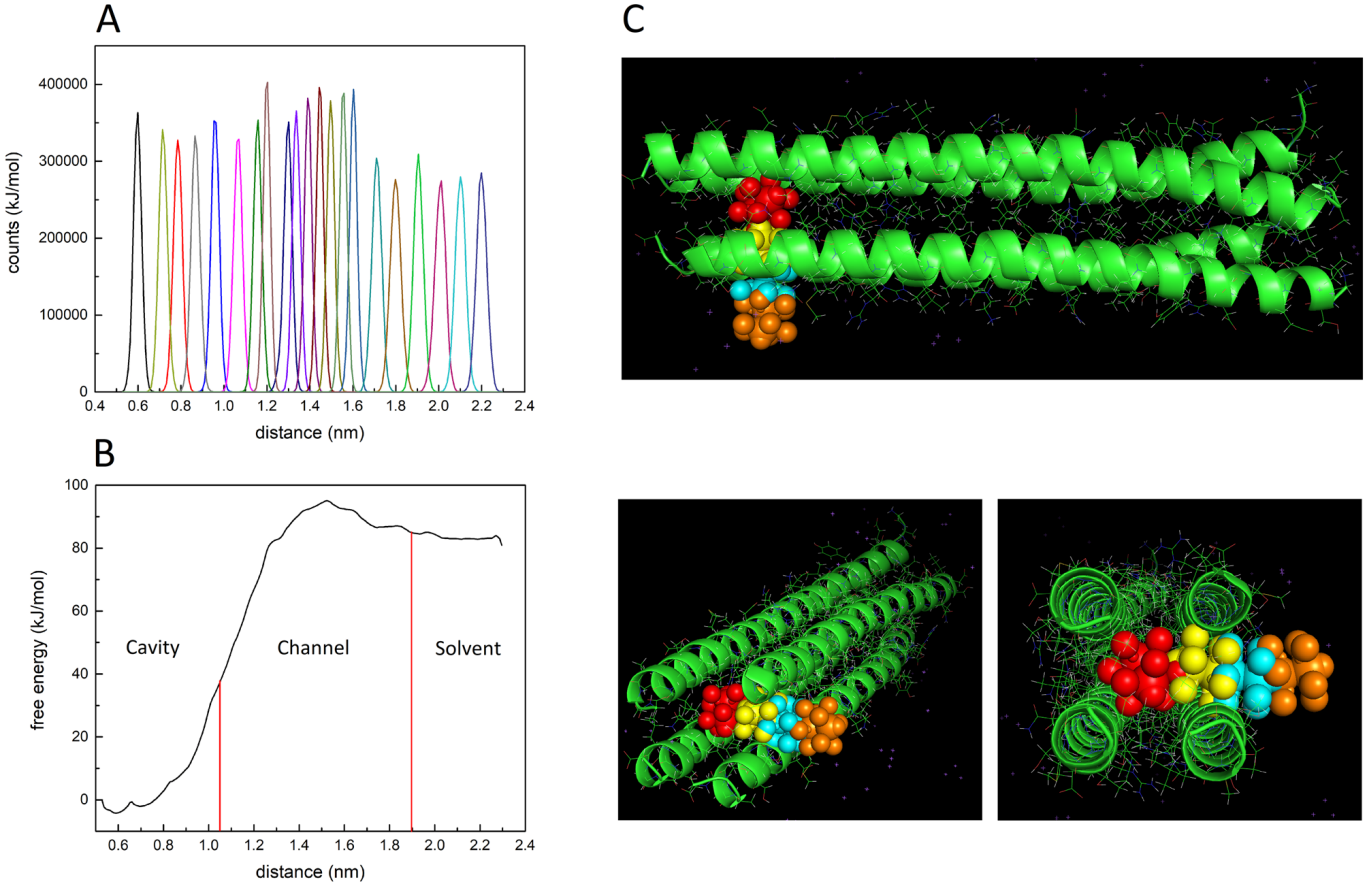
Molecular dynamics simulations of the exit pathway of *o*-carborane, C_2_B_10_H_12_ out of the C-terminal cavity of RHCC-NT. (**A**) Biased distribution, $P_{i}^{b} \left( d \right)$ of the simulation and (**B**) global free energy, *G*(*d*) as *o*-carborane moves out of the cavity through the interhelical channel into the solvent. (**C**) Cartoon representation of calculated *o*-carborane positions as it moves between the solvent and cavity through the interhelical channel of the nanotube. Protein figures were made using PyMOL 2.3.2 (https://pymol.org/2/).

The hydrophobic pockets of the tetrameric structure of RHCC-NT, which are mainly constructed by the side chains of leucine and isoleucine amino acids, allow for hydrophobic coordination of the *o*-carborane molecules (Fig. 1C). Upon expression of the protein nanotube, the cavities are filled with highly ordered water clusters which causes the system to be marginally energetically favourable. Thus, transfer of *o*-carborane into the hydrophobic cavities and displacement of the water clusters is energetically favourable due to the hydrophobic effect. The pathways and energetics of *o*-carborane transfer between one of the cavities of RHCC-NT and the solvent were investigated with molecular dynamics simulations using the methods of steered molecular dynamics^26–30^ and umbrella sampling^31–33^. The *o*-carborane in the C-terminal domain cavity was pulled out of the nanotube at a constant speed of *v* = 0.01 nm/ps. The boron cluster moved through a gap between two neighbouring protein helices along a nominally linear path transverse to the central channel by a moving harmonic steering force with spring constant *k* = 4000 kJ mol^−1^ nm^−2^ which was applied to the centre of mass of the molecule. We then used MD steering simulation to extract a large number of approximately equally spaced reference positions for the *o*-carborane along the exit trajectory. The method of Umbrella Sampling^31–33^ was used to construct a biased probability distribution $P_{i}^{b} \left( d \right)$ (Fig. 2A) and a free energy profile *G*_*i*_(*d*) over a sampling window centred on each reference position *i* as a function of the distance *d* along the exit pathway. This allowed us to generate a global free energy profile *G*(*d*) for the complete exit pathway by an application of WHAM^31,35^ (Fig. 2B). Inspection of the free energy profile shows that occupancy of the cavity by *o*-carborane is significantly favoured over the solvated state with $G_{sol} - G_{cav}$ ~ 80–100 kJ mol^−1^. Further, the activation barrier for the diffusion of *o*-carborane through the walls of RHCC-NT from the solvent to the cavity is much lower than the activation barrier for escape from the cavity with ${\Delta }G_{capture} \sim 10 - 20 \,{\text{kJ}}\,{\text{mol}}^{ - 1} < < {\Delta }G_{release} \sim 80 - 120\,{\text{kJ}}\,{\text{mol}}^{ - 1}$ (Table S3). Presumably, this is due to the different chemical environment that the *o*-carborane is placed in where the van der Waals interactions in the cavity increase its overall stability.

The molecular dynamics simulations allowed us to visualize the exit pathway along the steering trajectory at selected points (Fig. 2C). Over the interval, 0 ≤ *t* < 2500 ps the carborane molecule remained trapped in cavity. However, between 2500 ps < *t* < 8300 ps, the o-carborane molecule passed through the wall by bending residue side chains first in the vicinity of residue Glu38 (2500 ps < *t* < 4500 ps) and then in the vicinity of residue Arg33 (4500 ps < *t* < 8300 ps) before entering the solvent for *t* > 8300 ps. The movement between the cavity and the solvent created an interhelical channel where the backbone and the amino acid side chains of two of the four helices underwent large conformational changes. Nonetheless, due to the high stability of the nanotube with its many intramolecular interactions, the system did not fall apart.

The high stability of RHCC-NT in complex with *o*-carborane was confirmed through a thermal unfolding and size distribution analysis over a temperature range ranging from 20 to 95 °C. Over this temperature range, the internal fluorescence due to the Tyr residues did not show an unfolding event (Figure S2) and the cumulant radius remained the same (Figure S3). The polydispersity indices for the RHCC-NT and RHCC-NT-*o-*carborane were 0.232 and 0.347, respectively. This data indicates high stability of the RHCC-NT and a minimal change in dispersity upon uptake of the ligand.

### Eukaryotic cells internalize RHCC-NT

**Figure 3:**
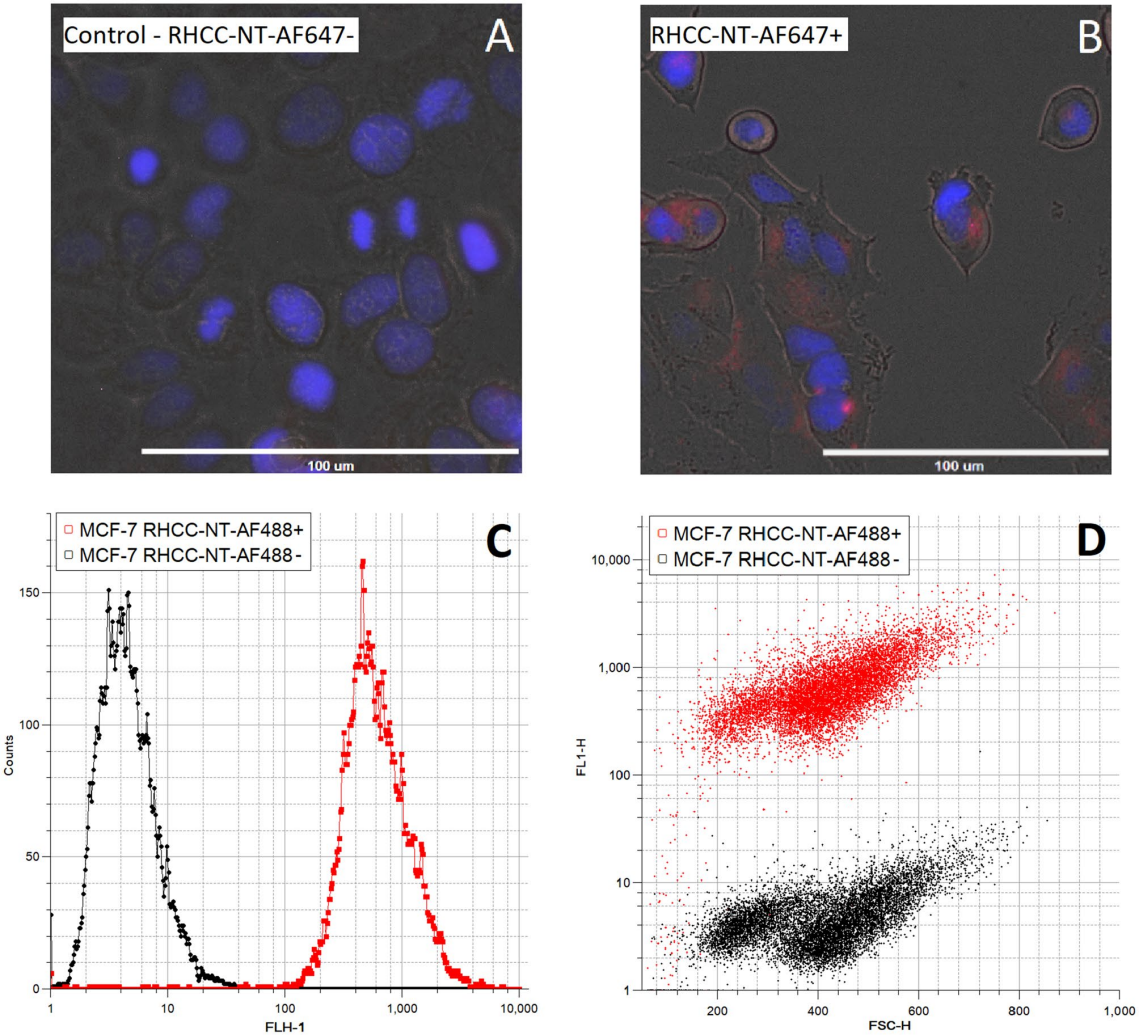
Nanotube cell-internalization assays using fluorescently labeled RHCC-NT show the accumulation of RHCC-NT inside of eukaryotic cells. (**A**) MCF-7 cells and (**B**) MCF-7 cells incubated with RHCC-NT-AF647 for 16 h were washed and imaged using multiple fluorescent and bright-field filters. Cell nuclei were stained with NucBlue™. (**C**) Fluorescent shift in flow cytometry of MCF-7 cells incubated with RHCC-NT-AF488 (red) compared against the negative control (black). (**D**) Forward scattering profile against the fluorescent shift of MCF-7 cells incubated with and without RHCC-NT-AF488.

The protein nanotube can be internalized by eukaryotic cells and distributes itself in the cytoplasm such as in MCF-7 cancer cells. To demonstrate this ability, we fluorescently labeled and exposed the nanotube to the cells for a set time after which the cells were washed, cell nuclei were DAPI-stained and imaged (Fig. 3A, B). The results produced fluorescent signals corresponding to the tagged protein inside the cells, clustered around the nucleus. Furthermore, the fluorescent intensities were measured using a flow cytometer to further confirm cellular uptake of RHCC-NT and determine the fraction of fluorescent shifted cells. Here, we found that the cell samples exposed to RHCC-NT showed a clear fluorescent intensity, FLH-1 shift from a median of 4.26 to 557.31, validating the uptake of the fluorescently labeled protein during the incubation period (Fig. 3C). CV values for the MCF-7 cell control and MCF-7 cells incubated with RHCC-NT were 77.45 and 75.35, respectively. Due to the significant fluorescent signal separation of the two cell populations, we were able to calculate the fraction of cells that were associated with RHCC-NT; based on a confidence interval of three standard deviations, 99.5% of the MCF-7 cell population that was incubated with the nanotube is above the threshold of association. Similarly, controlling for potential cell population changes, we compared the forward scattering against the fluorescent signal at 515/30 nm; whereas the fluorescent signal shifted, the forward scattering medians are statistically identical over 10,000 events; values measured were 413 (CV = 30.74) for the control and 416 (CV = 27.12) for the RHCC-NT exposed cell population (Fig. 3D). Thus, cell sizes remained unaffected upon exposure of the nanotube, indicating intact cell compartmentalization upon internalization of fluorescently labeled nanotube material.

## Discussion

### Nanotube takes up carborane for BNCT

BNCT is a compelling therapy used to fight several cancer types such as breast, head and neck carcinomas^1,8^. However, effective delivery of boron rich compounds, which is necessary for a potent treatment, has not been achieved and continues to be a research priority^3,9,36^. RHCC-NT provides a novel route for boron drug delivery based on its ability to take up *o*-carborane, a boron rich molecule. Molecular uptake was confirmed by boron NMR studies and by structural studies through crystallography, which allowed for chemical characterization of the protein–ligand complex. In the case of *o*-carborane, the ligand is stabilized in the center of the cavities through hydrophobic interactions with amino acid side chains which cause the entry of *o*-carborane to be energetically favourable. The uptake occurs spontaneously due to the hydrophobic effect which seeks to replace the native and energetically unstable water molecules present in the cavities. Chemical shifts in NMR demonstrated that *o*-carborane shifted from a hydrophilic to a hydrophobic environment in the presence of the protein nanotube. Mechanistically, MD simulations show that interhelical channels to the cavities form thus allowing *o*-carborane to move into the nanotube. Importantly, these intermediate channels do not seem to disturb the final overall shape and structure of the *o-*carborane bound nanotube. This same effect has previously been observed for other hydrophobic molecules taken up by RHCC-NT^15,16^.

Moreover, due to the large buried surface area (> 50%) of each nanotube helix and a high number of intramolecular ionic interactions, the nanotube is extremely stable which is evident by its high T_M_ of > 90 °C^13,37^. Our thermal size distribution analysis of the RHCC-NT in complex with the ligand did not show a thermal unfolding event. Although the polydispersity of the sample increased, the cumulant radius remained the same over the temperature gradient. This highly stable tetrameric structure prevents the protein from dissociating or exposing the hydrophobic cavities to the exterior. The significant stability confers the advantage that the drug of interest, in our case *o*-carborane, is buried inside the cavities; the hydrophobic molecules will not diffuse out of the carrier until the protein is denatured or digested. Additionally, the nanotube overcomes the hydrophobicity issue of *o*-carborane delivery into cells by increasing the effective solubility while completely shielding any possible toxic effects of the drug.

It is advantageous to deliver high boron quantities to target cells for BNCT as certain quantity thresholds have to be met in order for the treatment to be successful. Desired cellular boron quantities have been determined to be at around 10^9 10^B atoms/cell or 20 µg ^10^B/g tumour^1,5,11^. Exceeding these amounts per cell will increase the impact of neutron radiation and may positively affect final treatment results. However, sufficient boron delivery is a current challenge faced in BNCT development. Nanoparticle based drug delivery could assist in delivering such a payload to tumor cells, making BNCT a more compelling treatment option. The nanotube has a length of approximately 7.7 nm and a diameter of 1.8 nm, binding three *o*-carborane molecules. Assuming that 30 ^10^B atoms are integrated per RHCC-NT, adequate delivery would be achieved by delivering 1.5 mg RHCC-NT-C_2_B_10_H_12_/g tumour intracellularly.

### RHCC-NT as a drug delivery device

The proteinaceous nanotube RHCC-NT has significant potentials as a drug delivery device where the drug of interest is placed into the cavities and taken up by cells in close proximity. To our knowledge, there currently is only one protein based nanoparticle carrier clinically approved for cancer therapies; the nanoparticle albumin-bound paclitaxel^7,38^. Nanoparticles offer certain advantages over other methods, such as increased delivery, as well as stability and control of potentially toxic small molecules^7^. This is especially true for small molecules in cancer therapies such as paclitaxel where delivery and side effects are major obstacles for effective treatment. In parallel, neutron capture therapy is poorly utilized in treatment, as one of the main problems is the effective delivery of high avidity compounds like ^10^B isotopes. Naturally, boron compounds display low toxicity to the human body^3,39^ but delivery and targeting of boron rich compounds has been difficult to achieve due to either low and unspecific incorporation or high hydrophobicity such as in *o*-carborane molecules^40^. RHCC-NT in complex with *o*-carborane addresses these issues and provides a solution to this longstanding problem in the BNCT field.

As previous research has shown, RHCC-NT will bind surrounding FaDu eukaryotic cells in as little as 10 min and is incorporated intracellularly at significant levels at around 4 h^18^. These studies also showed that endotoxin-purified RHCC-NT only causes a minimal immune response in in vivo experiments; CD8^+^ T-cell production increased slightly but without a significant antibody response^18^. Our studies further explored effects in cancer cell lines and observed similar results where the nanotube is internalized by an entire cell population and accumulates in the cytoplasm around the nucleus. For this delivery system, cell internalization is not necessary as the mere attachment of the drug filled nanotubes to the target cells would allow for effective killing upon neutron exposure. However, efficient uptake of the nanotube is still desirable, as neutron therapy results improve when ^10^B is closer to the cell’s nucleus as DNA damage from free radicals and ionizing radiation is most effective in initiating cell death^8,41^. Additionally, release of *o*-carborane from the nanotube is not necessary since neutron beams would penetrate the protein structure inside of a cell causing it to activate its desired effects. Thus, *o*-carborane loaded nanotubes in combination with intravenous injections could prove to be a compelling BNCT drug delivery method. Future studies should aim to explore the nanotube’s in vivo drug delivery distribution and accumulation for BNCT.

As these proteins are highly modular, there are additional possibilities for attaching biomolecular tags that would allow the specific delivery of the nanotube to target cells. Certain cell receptors such as vascular endothelial growth factor (VEGFR) or epidermal growth factor receptor (EGFR) which are overexpressed in cancer cells, offer potential targets for nanotube modifications^3,42^. Previous research has also focused on attaching folate molecules to nanoparticles for cancer therapy development which offers the advantage of targeting overexpressed folate receptors on tumor cells^43,44^. In the case of RHCC-NT, functionalizing the nanotube is a possibility that warrants further investigation.

The physicochemical characterization of RHCC-NT as a drug delivery device presented in this study, specifically for BNCT, highlights its many advantageous properties for the delivery of hydrophobic molecules such as *o*-carborane. Although further investigations have to prove its viability for targeting and elimination of cancer cells as a result of delivery and neutron activation of boron clusters, the nanotube shows promising abilities as a nanoparticle based therapeutic approach for various carcinomas.

## Supplementary Information

Supplementary Information.

## Data Availability

Electron densities and atomic coordinates for the RHCC in complex with o-carborane structure is available at the Protein Data Bank (PDB ID 7R6H). Additional data is available in the supplemental material and upon reasonable request.
