# Supplementary material for: Boron rich nanotube drug carrier system is suited for boron neutron capture therapy
Source: Sci Rep. 2021 Jul 30;11:15520. doi: 10.1038/s41598-021-95044-0 (PMC8324832; doi:10.1038/s41598-021-95044-0)
Supplement: Supplementary file 1 — Supplementary Information. [file 41598_2021_95044_MOESM1_ESM.pdf]

Boron Rich Nanotube Drug Carrier System Is Suited for Boron Neutron Capture Therapy – Supplemental Information

***Fabian Heide [a]** - Department of Chemistry, University of Manitoba, Winnipeg, MB R3T 2N2, Canada

Matthew McDougall [a] - Department of Chemistry, University of Manitoba, Winnipeg, MB R3T 2N2, Canada

Candice Harder-Viddal - Department of Chemistry and Physics, Canadian Mennonite University, Winnipeg, MB R3P 2N2, Canada

Roy Roshko - Department of Physics and Astronomy, University of Manitoba, Winnipeg, MB R3T 2N2, Canada

David Davidson - Department of Chemistry, University of Manitoba, Winnipeg, MB R3T 2N2, Canada

Jiandong Wu - Institute of Biomedical and Health Engineering, Shenzhen Institutes of Advanced Technology, Chinese Academy of Sciences, Shenzhen 518055, China

Camila Aprosoff - Department of Chemistry, University of Manitoba, Winnipeg, MB R3T 2N2, Canada

Aniel Moya-Torres - Department of Chemistry, University of Manitoba, Winnipeg, MB R3T 2N2, Canada

Francis Lin - Department of Physics and Astronomy, University of Manitoba, Winnipeg, MB R3T 2N2, Canada

***Jörg Stetefeld** - Department of Chemistry, University of Manitoba, Winnipeg, MB R3T 2N2, Canada

*Corresponding Authors:

Jörg Stetefeld - +1(204)474-9731, Jorg.Stetefeld@umanitoba.ca

Fabian Heide - +1(204)332-0853, Heidef@myumanitoba.ca

[a] These authors have contributed equally and share first authorship.

Crystallographic Data Table

Table S1. Crystallographic data table of RHCC-NT in complex with *o*-carborane (PDB ID 7R6H).

	RHCC-NT o-carborane
Wavelength	1.542
Resolution range	18.57 - 2.2 (2.279 - 2.2)
Space group	P 21 21 21
Unit cell	34.45 55.92 110.23 90 90 90
Total reflections	22424 (2218)
Unique reflections	11221 (1110)
Multiplicity	2.0 (2.0)
Completeness (%)	98.01 (98.66)
Mean I/sigma(I)	14.97 (8.33)
Wilson B-factor	21.6
R-merge	0.02599 (0.06346)
R-meas	0.03676 (0.08975)
R-pim	0.02599 (0.06346)
CC1/2	0.999 (0.994)
*CC**	1 (0.999)
Reflections used in refinement	11174 (1105)
Reflections used for R-free	826 (84)
R-work	0.2217 (0.2502)
R-free	0.2666 (0.2782)
CC(work)	0.966 (0.922)
CC(free)	0.898 (0.836)
Number of non-hydrogen atoms	1720
macromolecules	1625
ligands	72
solvent	59
Protein residues	205
RMS(bonds)	0.003
RMS(angles)	0.42
Ramachandran favored (%)	100
Ramachandran allowed (%)	0
Ramachandran outliers (%)	0
Rotamer outliers (%)	0.54
Clashscore	3.9
Average B-factor	35.23
macromolecules	35.26
ligands	30.11
solvent	37.63
Number of TLS groups	4

Detailed Materials and Methods

Molecular Dynamics Simulations

Molecular dynamics simulations were performed with the GROMACS molecular dynamics simulation package [1] using the AMBER force field parm94, and the TIP3P water model. All simulations were performed on a RHCC-NT-$C_2B_{10}H_{12}$ complex in which all four cavities were simultaneously occupied by a single carborane molecule. The complex model was prepared by inserting the ligand into each cavity of the measured structure of the RHCC-NT (PDB: 1FE6) [2], crystallized at T = 298 K. Force field parameters (Amber 99) for *o*-carborane were obtained from Sarosi et al. [3] which were adapted and optimized for bond lengths, torsion parameters and bonded terms using GAFF values by Timofeeva et al. [4] and Gamba et al. [5]. The partial charges for the ligand were generated using Gaussian electrostatic potential fitting and simultaneously calculated at the HF/6-31+G* level. Long-range electrostatics were employed through the Ewald particle mesh method with a non-bonded cut-off range of 1.0 nm.

Table S2. Molecular Dynamics simulation parameters.

Parameter	Value
Solvation Box	5.2 nm x 5.2 nm x 10.4 nm
SPC water molecules	8560
Ions	16 Na+
$E_{min, initial}$[1]	1000 kJ/mol
$E_{min, convergence}$[1]	40-50 kJ/mol
Pressure	1 atm
Temperature[2]	300 K
Time[2]	2 ns

[1] *Energy minimization was achieved through the method of steepest descent.*

[2] *Parameters during production run using a Berendsen barostat and a velocity-rescaling thermostat.*

Results

Table S3. Standard free energies[1] in kJ/mol to transfer $C_2B_{10}H_{12}$ from solvent to the four cavities of RHCC-NT.

Cavity	$\Delta G_{cav \rightarrow gas}{}^{2}$	ΔG_r^0	$\Delta G_{sol \rightarrow gas}{}^{2}$	$\Delta G_{trans} = \Delta G_{sol \rightarrow gas} - \Delta G_{cav \rightarrow gas} - \Delta G_r^0$
1	$+16.9 \pm 2.7$	-1.5	$+8.7 \pm 0.8$	-6.7 ± 3.5
2	$+33.2 \pm 1.1$	-1.5	$+8.7 \pm 0.8$	-23.0 ± 1.9
3	$+22.5 \pm 0.7$	-1.5	$+8.7 \pm 0.8$	-12.3 ± 1.5
4	$+19.1 \pm 1.3$	-1.5	$+8.7 \pm 0.8$	-8.9 ± 2.1

[1] *Free energies were calculated using the method of double-decoupling with a flat bottom harmonic well (FBHW) restraint potential* [6,7].

[2] *Values were computed using the multi-configurational thermodynamic integration (MCTI) method* [6,7].

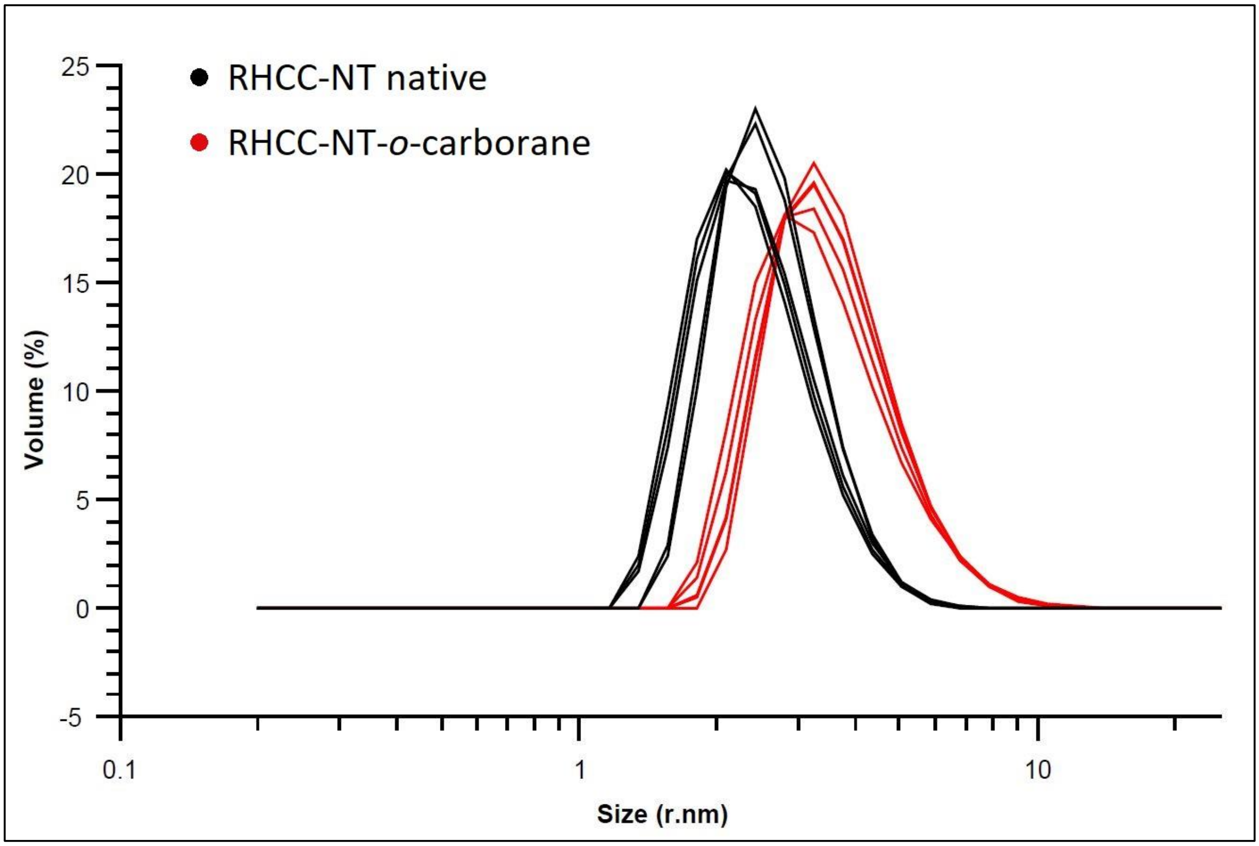

Figure S1. Size distribution profile of native RHCC-NT (black) and *o*-carborane incubated RHCC-NT (red). The hydrodynamic radius is shifted slightly indicating ligand uptake and possible coiled coil conformational changes.

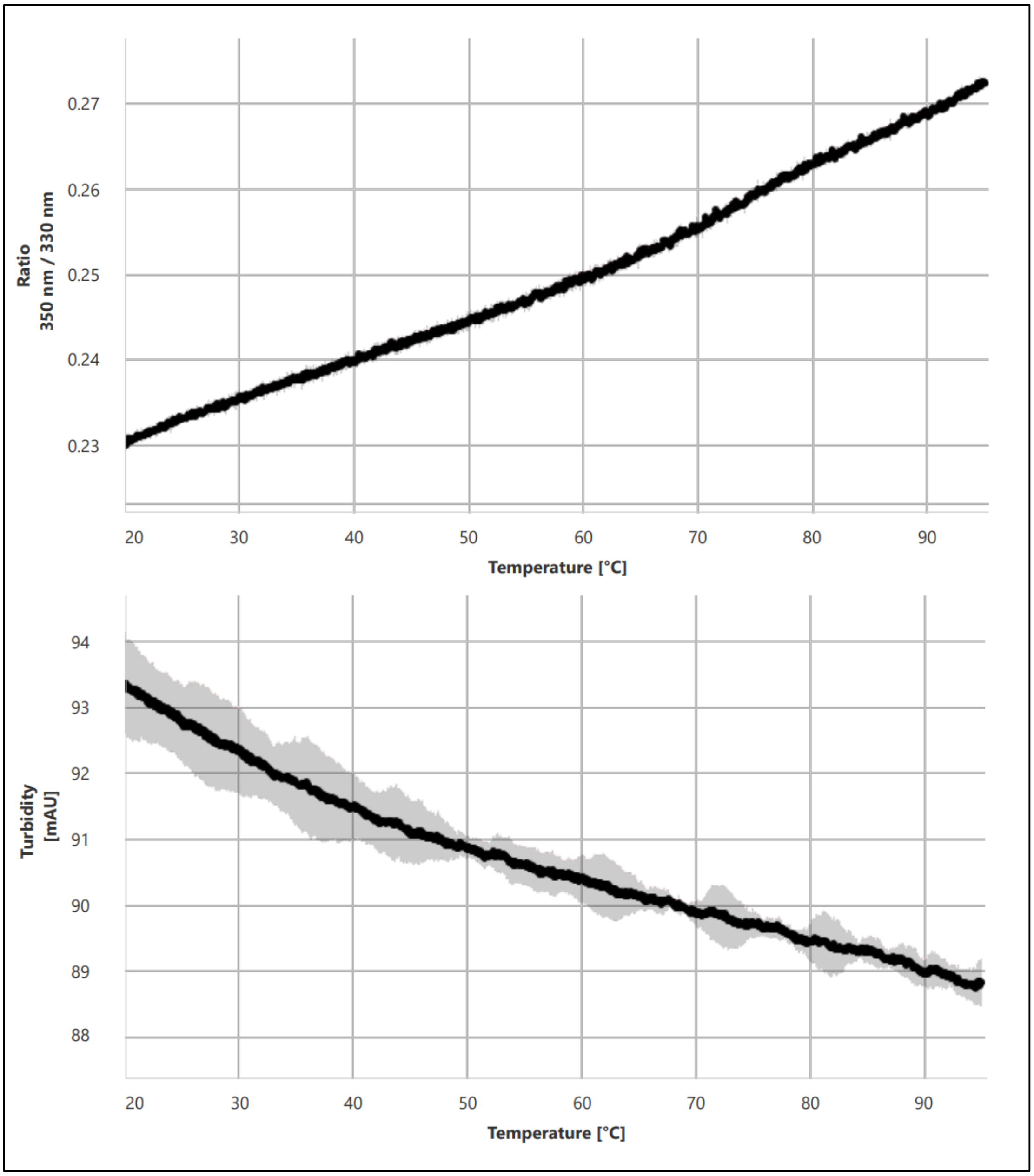

Figure S2. Thermal unfolding analysis of RHCC-NT containing *o*-carborane. The 350 nm/ 330 nm ratio, which measures the internal Trp/Tyr fluorescence, does not show a shift from a folded to an unfolded state. In addition, turbidity measurements do not show any large changes in aggregation states of the sample, indicating a folded state of the RHCC-NT across the temperature gradient. Protein samples were measured in triplicates.

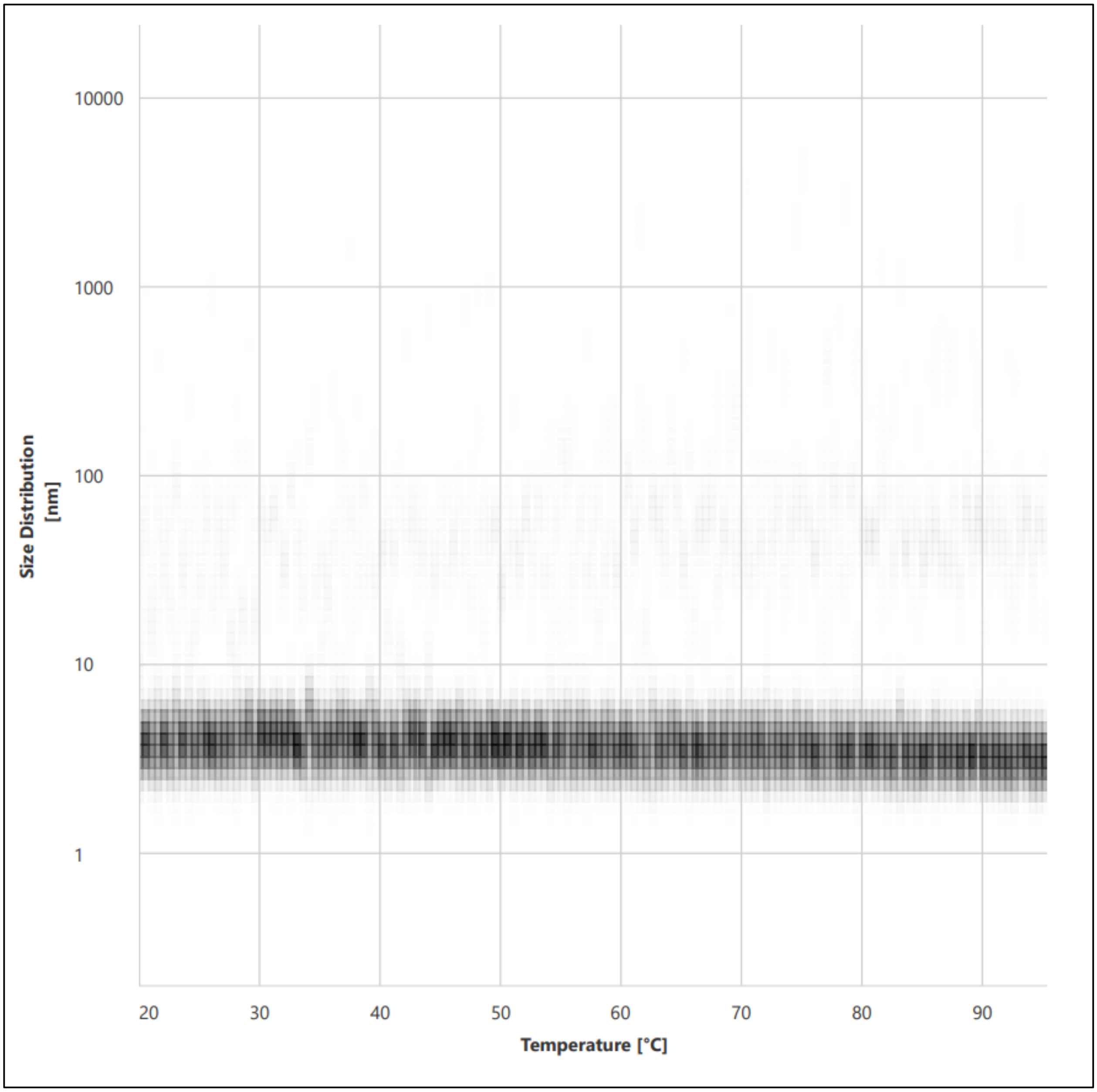

Figure S3. Size distribution analysis of RHCC-NT containing *o*-carborane over a temperature gradient. The size distribution of RHCC-NT does not change with increasing temperature, indicating a stable and intact nanotube structure. Samples were measured in triplicates.

References

(1) Van Der Spoel, D.; Lindahl, E.; Hess, B.; Groenhof, G.; Mark, A. E.; Berendsen, H. J. C. GROMACS: Fast, Flexible, and Free. *J. Comput. Chem.* **2005**, *26* (16), 1701–1718. https://doi.org/10.1002/jcc.20291.

(2) Stetefeld, J.; Jenny, M.; Schulthess, T.; Landwehr, R.; Engel, J.; Kammerer, R. A. Crystal Structure of a Naturally Occurring Parallel Right-Handed Coiled Coil Tetramer. *Nat. Struct. Biol.* **2000**, *7* (9), 772–776. https://doi.org/https://doi.org/10.1038/79006.

(3) Sárosi, M.-B.; Lybrand, T. P. Molecular Dynamics Simulation of Cyclooxygenase-2 Complexes with Indomethacin Closo -Carborane Analogs. *J. Chem. Inf. Model.* **2018**, *58* (9), 1990–1999. https://doi.org/10.1021/acs.jcim.8b00275.

(4) Timofeeva, T. V.; Suponitsky, K. Y.; Yanovsky, A. I.; Allinger, N. L. The MM3 Force Field for 12-Vertex Boranes and Carboranes. *J. Organomet. Chem.* **1997**, *536–537*, 481–488. https://doi.org/10.1016/S0022-328X(96)06819-2.

(5) Gamba, Z.; Powell, B. M. The Condensed Phases of Carboranes. *J. Chem. Phys.* **1996**, *105* (6), 2436–2440. https://doi.org/10.1063/1.472111.

(6) Gilson, M. K.; Given, J. A.; Bush, B. L.; McCammon, J. A. The Statistical-Thermodynamic Basis for Computation of Binding Affinities: A Critical Review. *Biophys. J.* **1997**, *72* (3), 1047–1069. https://doi.org/10.1016/S0006-3495(97)78756-3.

(7) Hamelberg, D.; McCammon, J. A. Standard Free Energy of Releasing a Localized Water Molecule from the Binding Pockets of Proteins: Double-Decoupling Method. *J. Am. Chem. Soc.* **2004**, *126* (24), 7683–7689. https://doi.org/10.1021/ja0377908.
